# *In vitro* evaluation of stability and hemostatic efficacy of single-donor lyophilized canine plasma

**DOI:** 10.3389/fvets.2025.1663953

**Published:** 2025-11-03

**Authors:** Sumin Cha, Chaewon Shin, Changkeun Kang, Dong-In Jung, Kyu-Woan Cho, Hyeona Bae, Dohyeon Yu

**Affiliations:** College of Veterinary Medicine, Gyeongsang National University, Jinju, Republic of Korea

**Keywords:** coagulation, dog, lyophilized plasma, plasma, transfusion

## Abstract

**Introduction:**

Interest in lyophilized plasma products has increased. However, data on their use in dogs are limited. This study aimed to evaluate the *in vitro* stability and hemostatic efficacy of single-donor lyophilized canine plasma.

**Methods:**

Ten canine plasma units were lyophilized and stored at −80 °C, 4 °C, room temperature, and 38 °C for 45 days. The plasma compositions before and after lyophilization were compared to assess the impact of the lyophilization. The following parameters were assessed to evaluate storage stability: blood gas analysis, biochemical parameters, coagulation profiles [prothrombin time (PT); activated partial thromboplastin time (aPTT); fibrinogen concentration; the activities of coagulation factors II, V, VIII, IX, X, and XII, as well as those of antithrombin (AT); and protein C], and kaolin-activated thromboelastography. Aerobic bacterial cultures were performed using thioglycollate broth to assess sterility. Lyophilized plasma samples were reconstituted to 50, 60, 80, and 100% of the original plasma volume to assess the effects of different reconstitution volumes on plasma components. Total protein, albumin, osmolality, selected coagulation factors (II and V), fibrinogen, and AT were measured and compared across the reconstituted groups.

**Results:**

Lyophilization decreased the partial pressure of carbon dioxide and increased the pH. No other significant immediate changes were observed. Plasma stored at −80 °C and 4 °C maintained stable biochemical and coagulation profiles over 45 days of storage, with only a slight but statistically significant decrease in fibrinogen concentrations on Days 30 and 45 for refrigerated conditions when compared with post-lyophilization values. Significant reductions in the activities of coagulation factors II, V, and VIII were observed at room temperature by Day 45, whereas PT, aPTT, and thromboelastography remained within normal reference ranges relative to the post-lyophilization values. Storage at 38 °C led to marked deterioration in coagulation function, as evidenced by the prolonged PT and aPTT, substantial decline in fibrinogen concentrations, and >50% reduction in the activity of all assessed coagulation factors relative to the post-lyophilization values. The AT activity declined for all storage groups, whereas protein C and thromboelastography profiles remained relatively stable, except at 38 °C. No bacterial growth was observed in any reconstituted plasma samples across all storage temperature and time points. Reconstitution at lower volumes (50 and 60%) increased the concentrations of albumin and activities of coagulation factors and osmolality.

**Conclusion:**

The lyophilization process did not significantly affect the concentrations or activities of major plasma proteins, including coagulation factors and anticoagulant proteins. Storage at −80 °C and 4 °C for 45 days preserved the stability of biochemical and hemostatic parameters. However, storage at room temperature resulted in minor reductions in select coagulation factors (such as factors II, V, and VIII). PT, aPTT, and thromboelastography parameters remained within the normal reference ranges, confirming the short-term stability of the product. Changes in the reconstitution volume affected plasma concentration and highlighted the potential of lyophilized plasma as a rapid resuscitative product in veterinary medicine.

## 1 Introduction

Plasma products are frequently used for patients with critical illness due to their essential roles in supporting coagulation and hemostasis. They include fresh plasma, fresh frozen plasma (FFP), frozen plasma, and cryoprecipitate based on their method of preparation and storage conditions. Despite their therapeutic benefits, traditional plasma products have several limitations, including strict storage conditions to preserve protein stability, time-consuming preparation procedures, and the risk of volume overload with excessive administration.

Recent efforts have been directed toward developing novel plasma products to overcome the limitations of traditional products. Among these, lyophilized plasma (LP) has emerged as one such promising alternative. LP has several advantages over traditional plasma products, including room-temperature storage, high portability, and rapid reconstitution. It can also be formulated as a hyperosmotic and hyperoncotic solution, which may extend its application to low-volume resuscitation strategies (1, 2). However, its clinical adoption remains limited due to the lack of comprehensive data on its biochemical stability, safety, and efficacy. The risk of pathogen transmission also remains a concern for all blood-derived products. Several studies have evaluated the effects of storage conditions on LP stability in human medicine, with findings indicating that stability can be preserved for 6 months to 2 years depending on the specific storage temperature and duration (3–7). While a study has explored the stability of reconstituted LP in dogs (8), these have primarily focused on post-reconstitution properties. To date, no study has systemically investigated the intrinsic stability of the lyophilized product itself or the impact of storage conditions prior to reconstitution.

This study aimed to evaluate the effects of lyophilization on the biochemical and coagulation profiles of canine plasma and to assess the *in vitro* stability and potential clinical utility of single-donor canine lyophilized plasma. Based on this aim, the following hypotheses were evaluated: (1) the lyophilization process would not significantly alter plasma components; (2) LP stability would vary depending on storage conditions, including temperature and duration; and (3) the reconstitution volume would lead to volume-dependent changes in plasma component concentrations.

## 2 Materials and methods

### 2.1 Study design

**Figure 1:**
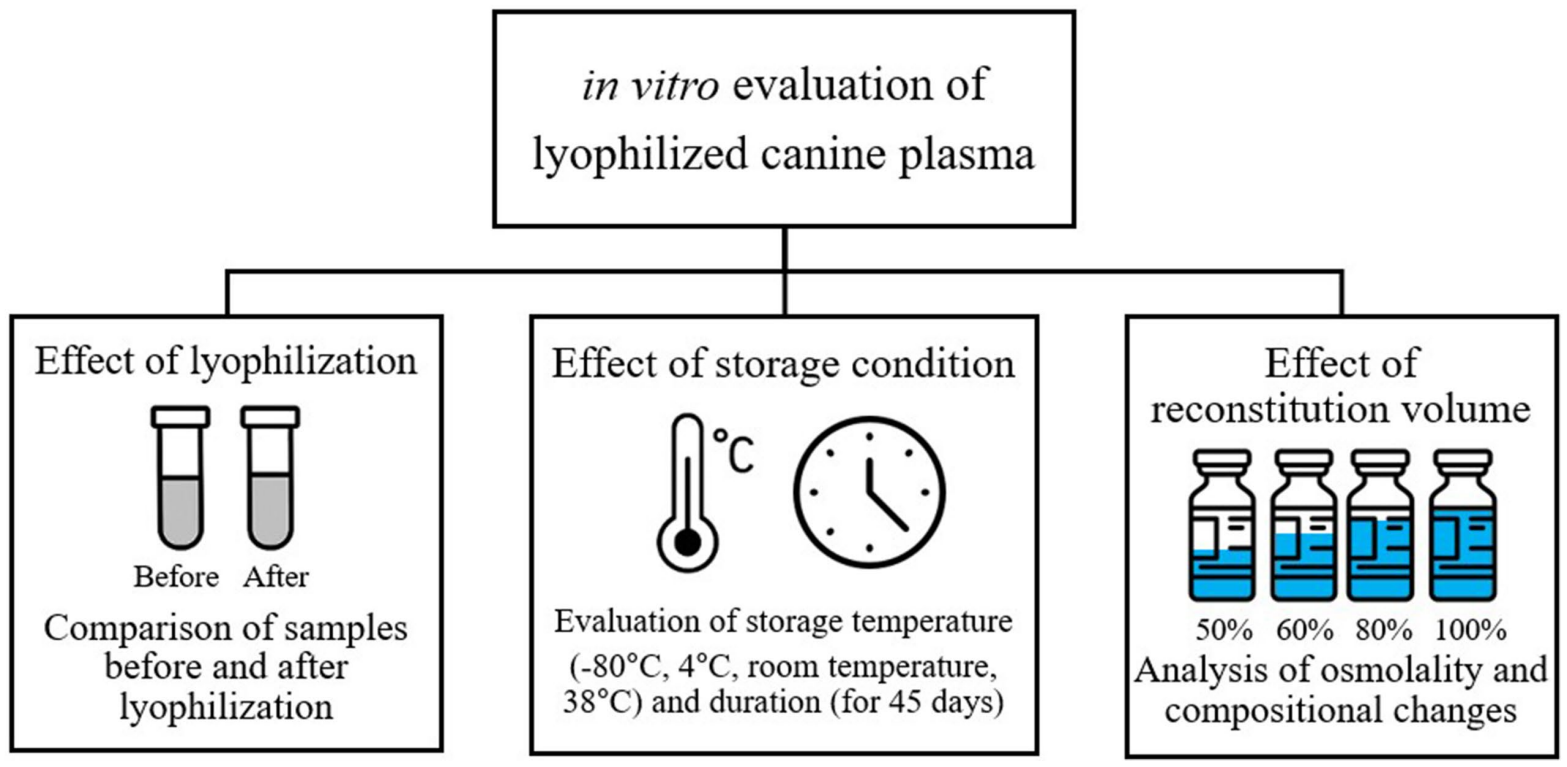
Study design for *in vitro* evaluation of lyophilized canine plasma stability and clinical applicability.

Three *in vitro* experimental protocols were implemented to evaluate the stability and clinical applicability of LP. First, the effects on plasma composition were assessed by comparing samples before and after lyophilization. Second, the impact of storage temperature and duration on the stability of LP components was evaluated. Lastly, the influence of different reconstitution volumes on osmolality regulation and compositional changes was investigated (Figure 1). The research protocol was reviewed and approved by the Institutional Animal Care and Use Committee (IACUC) GNU-240129-D0031 of Gyeongsang National University.

### 2.2 Sample preparation

#### 2.2.1 Plasma collection

Ten units of fresh whole blood were collected from five healthy purpose-bred research Beagle dogs (weighing up to 15 kg) with normal hematologic and hemostatic profiles on two separate occasions, spaced at least 2 months apart. Approximately 10–15 mL/kg of blood was obtained via jugular venipuncture using a citrate–phosphate-dextrose-adenine (CPDA) anticoagulant multi-bag system (CY Medical Co., Ltd., Seoul, Korea), equipped with a 16-gauge needle, at a blood-to-anticoagulant ratio of 7:1. The venipuncture site was clipped and aseptically prepared using 70% isopropyl alcohol, and 2% lidocaine was administered intradermally to provide local anesthesia. Blood was then collected by gravity flow without suction to minimize hemolysis. Plasma was separated from refrigerated whole blood by centrifugation at 5,000 × *g* for 7 min at 4 °C within 4 h of collection, without prior leukoreduction. The separated plasma was then aliquoted into 22 individually sterilized commercial glass vials (HanSol Science, Seoul, Korea) in equal volumes using an open system with manual pipetting for subsequent analysis and processing. Each vial was designated for a specific storage condition (defined by temperature and duration) and reconstitution volume.

#### 2.2.2 Lyophilization

The stock solution containing glycine and citric acid was prepared to stabilize plasma proteins. Plasma samples were mixed with the stock solution at a 19:1 ratio, resulting in final concentrations of 60 mM glycine and 2 mM citric acid. The plasma-stabilizer mixture was stored at −80 °C for 7 days before lyophilization. Lyophilization was performed over 2 days using a bench-top freeze dryer (TFD8503, IlShinBioBase, Dongducheon, Korea) set at −80 °C and 5 mTorr to produce the final lyophilized canine plasma samples. Each glass vial was securely sealed with a sterile rubber stopper and an aluminum cap using a manual crimping tool (HanSol Science, Seoul, Korea), ensuring an aseptic condition for long-term storage.

#### 2.2.3 Storage

Immediately after lyophilization, LP samples were stored under four different temperature conditions: −80 °C, 4 °C, room temperature, and 38 °C. Storage at −80 °C was maintained using a deep freezer, while 4 °C storage was maintained in a laboratory refrigerator with adjustable settings. The 38 °C condition was maintained using a temperature-controlled incubator. All equipment was dedicated exclusively to this experiment to ensure temperature consistency and avoid external influence.

### 2.3 Analysis of plasma components

#### 2.3.1 Reconstitution

LP was reconstituted using sterile Water for Injection (JW Pharmaceutical, Seoul, Korea) at room temperature for subsequent analysis. Standard reconstitution was performed using 94% of the original plasma volume. Additional plasma vials were reconstituted to final volumes corresponding to 50, 60, 80, and 100% of the original volume to evaluate the effects of different reconstitution volumes. All samples were gently mixed using a rolling shaker for 10–15 min after reconstitution to ensure complete dissolution, which was confirmed visually by the absence of any visible precipitate. For analysis, the reconstituted samples were collected using a syringe via negative pressure aspiration.

#### 2.3.2 Evaluation parameters

The evaluation parameters specific to each experiment are summarized in Table 1. Blood gas parameters were measured using a blood gas analyzer (Stat Profile pHOX Ultra Analyzer; Nova Biomedical, Waltham, MA, USA). Osmolality was assessed using a vapor pressure osmometer (Model 5,600; ELITechGroup, Logan, UT, USA). Biochemical parameters such as the total protein, albumin, and glucose concentrations were analyzed using an automated chemistry analyzer (Catalyst Dx Chemistry Analyzer; IDEXX Laboratories, Westbrook, ME, USA) based on a dry-slide reflectance photometric assay system.

**Table 1:** Evaluation parameters used in each experiment.

Experiment	Purpose	Evaluation parameters
1	Effect of lyophilization	pH, partial pressure of carbon dioxide, total protein, albumin, osmolality, coagulation factors (II, V, VIII, IX, X, XII)
2	Effect of storage condition	Total protein, albumin, glucose, osmolality, PT, aPTT, fibrinogen, coagulation factors (II, V, VIII, XI, X, XII), antithrombin, protein C, TEG parameters (R time, K time, alpha angle, MA)
3	Effect of reconstitution volume	Total protein, albumin, osmolality, fibrinogen, coagulation factors (II, V), antithrombin

*Units: partial pressure of carbon dioxide = mmHg; total protein/albumin = g/dL; osmolality = mOsm/kg; coagulation factors = % activity; PT/aPTT = sec; fibrinogen = mg/dL; TEG parameters = standard TEG units.

*PT, prothrombin time; aPTT, activated partial thromboplastin time; TEG, thromboelastography; R time, reaction time; K time, clot formation time; MA, maximum amplitude.

An automated coagulation analyzer (Sta Compact Max; Diagnostica Stago, Asnières-sur-Seine, France) with commercially available reagents was used to assess coagulation profiles, including prothrombin time (PT), activated partial thromboplastin time (aPTT), fibrinogen concentration, and the activities of coagulation factors II, V, VIII, IX, X, and XII and the endogenous anticoagulants antithrombin and protein C. Fibrinogen concentration was measured using the Clauss method, and individual coagulation factor activities were determined using one-stage clotting assays with factor-deficient human plasma. Antithrombin and protein C were measured using chromogenic substrate assays. Calibration was performed using human plasma calibrators, and internal quality control was maintained through routine analysis of commercially available control plasmas, following the manufacturer’s protocols. Thromboelastography was performed at 37 °C using a Single TEG® 5,000 Hemostasis Analyzer System (Haemonetics Corporation, Braintree, MA, USA) with kaolin as the coagulation activator to evaluate clot formation kinetics and strength. In the kaolin tube, 400 μL of citrated whole blood from a dog with a normal hemostatic profile—held for 30 min prior to analysis—was mixed with 300 μL of lactated Ringer’s solution and 300 μL of the LP sample to prepare a total volume of 1,000 μL of diluted blood. Then, 340 μL of this diluted sample was added to 20 μL of 0.2 mM calcium chloride in a prewarmed disposable plastic cup, using manual pipetting as recommended for thromboelastography assays (9, 10). As only a single analyzer was available, measurements were performed sequentially. Therefore, multiple citrated blood samples were obtained from the same healthy donor dog at different time points, with a maximum holding time of 30 min before use. Calibration and quality control procedures were conducted according to the manufacturer’s instructions. The following thromboelastography parameters were recorded: reaction time (R), clot formation time (K), alpha angle, and maximum amplitude (MA). The analysis was continued until the MA value plateaued, reflecting stabilization of clot strength. For aerobic bacterial culture, 1 mL of reconstituted plasma collected from each storage temperature and time point was aseptically inoculated into 9 mL of sterile thioglycolate broth (Asan Pharmaceutical Co., Ltd., Hwaseong, Korea). The cultures were incubated at 38 °C for 48 h. Turbidity or visible sediment indicated bacterial growth, whereas their absence was considered a negative result.

### 2.4 Statistical methods

All data were analyzed using SPSS for Windows (version 27.0; SPSS Inc., Chicago, IL, USA) with non-parametric statistical methods. The Wilcoxon signed-rank test was used to compare values before and after lyophilization. The Friedman test was used to evaluate differences related to storage conditions (temperature and duration) and reconstitution volume. Dunn’s multiple comparisons test was used for *post hoc* analysis to determine pairwise differences between conditions when significant differences were detected.

All data are presented as medians with interquartile ranges (IQRs) and visualized using box plots, where boxes represent the 25th to 75th percentiles and whiskers indicate the minimum and maximum values. Statistical significance was defined as *p* < 0.05, with the following symbols used throughout the results to indicate significance levels: *p < 0.05, **p < 0.01, ***p < 0.001, and ****p < 0.0001. All comparisons were applied to the post-lyophilization (Day 0) and 100% dilution conditions. All graphical analyses were performed using GraphPad Prism version 8 (GraphPad Software, La Jolla, CA, USA).

## 3 Results

### 3.1 Effect of lyophilization on blood laboratory test results

The pH significantly increased after lyophilization (*p* = 0.02), which was attributed to a marked reduction in the partial pressure of carbon dioxide (*p* = 0.002), as shown in Table 2. In contrast, no significant changes were observed in total protein, albumin, glucose, osmolality, or the activities of coagulation factors II, V, VIII, IX, X, and XII.

**Table 2:** Effect of lyophilization on blood laboratory test results: comparison of plasma parameters before and after lyophilization.

Variables	pH	pCO2 (mmHg)	Total protein (g/dL)	Albumin (g/dL)	Osmolality (mOsm/kg)	Factor II (%)	Factor V (%)	Factor VIII (%)	Factor IX (%)	Factor X (%)	Factor XII (%)
Pre-lyophilization	7.32 (7.29–7.42)	27.15 (23.78–28.73)	4.25 (3.75–4.53)	2.40 (1.88–2.53)	314.0 (304.5–316.3)	99.5 (8.8–108.5)	943.5 (763.5–1,059)	320.5 (141.5–363.3)	102.5 (62.3–147.8)	143.0 (119.8–175.0)	53.5 (34.5–112.0)
Post-lyophilization	7.67** (7.59–7.78)	10.25** (8.35–11.08)	4.30 (3.57–4.33)	2.35 (2.05–2.50)	312.0 (301.3–321.0)	103 (86.5–118.3)	971.0 (731–1,323)	402.5 (218.5–478.3)	100 (80.8–141.8)	127.5 (120.0–154.3)	76.0 (50.5–108.0)

Data are presented as median (IQR). **p < 0.01 versus pre-lyophilization group.

### 3.2 Effect of storage temperature and duration on stability

The quantitative analysis of lyophilized plasma revealed changes in biochemical and coagulation parameters based on storage temperature and duration.

#### 3.2.1 Stability evaluation over 45 days of storage at −80 °C and 4 °C

**Figure 2:**
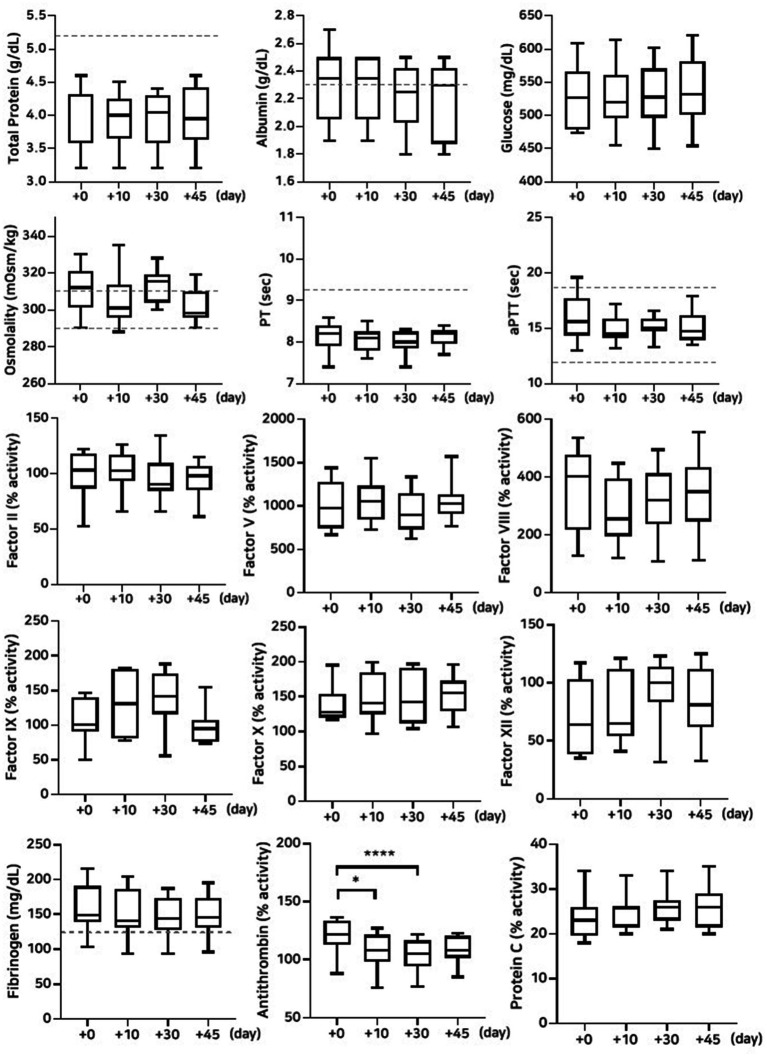
Analysis of changes in plasma composition over 45 days of storage at −80 °C (*n* = 10 samples per time point). Friedman test analysis of biochemical and coagulation parameters over 45 days of storage at −80 °C. The dotted lines represent the upper reference limit for PT, the upper and lower reference limits for osmolality and aPTT, and the lower reference limits for total protein, albumin, and fibrinogen concentrations. *p < 0.05, **p < 0.01, ***p < 0.001, and ****p < 0.0001.

**Figure 3:**
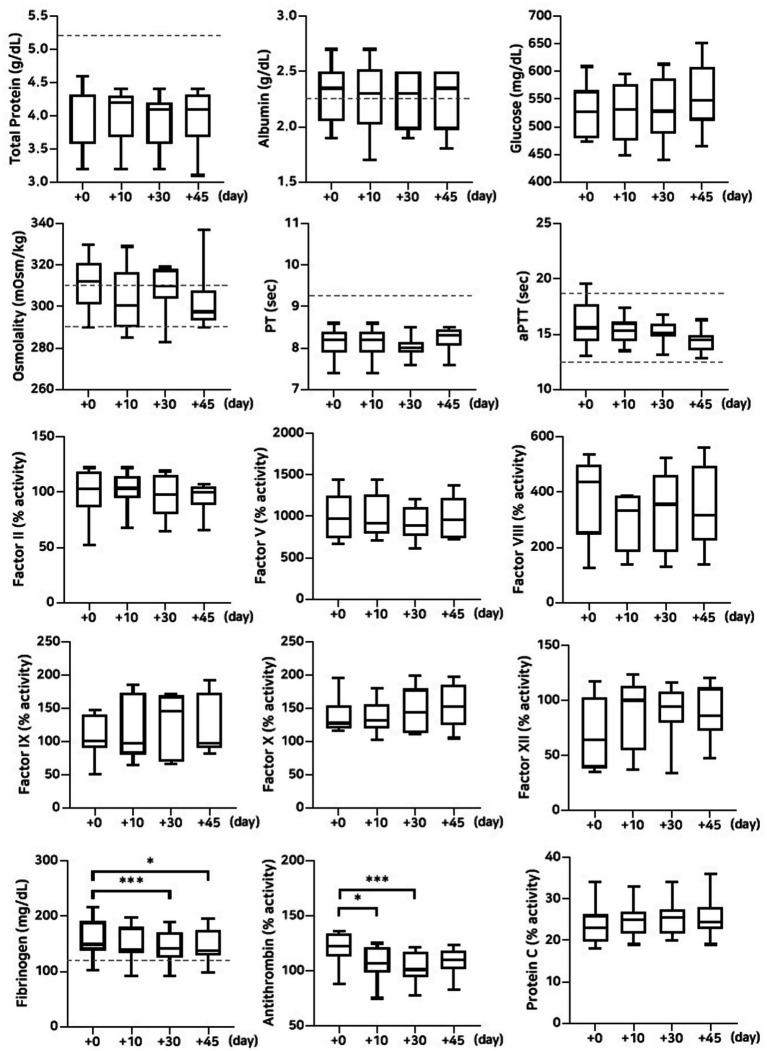
Analysis of changes in plasma composition over 45 days of storage at 4 °C (*n* = 10 samples per time point). Friedman test analysis of biochemical and coagulation parameters over 45 days of storage at 4 °C. The dotted lines represent the upper reference limit for PT, the upper and lower reference limits for osmolality and aPTT, and the lower reference limits for total protein, albumin, and fibrinogen concentrations. *p < 0.05, **p < 0.01, ***p < 0.001, and ****p < 0.0001.

The lyophilized plasma maintained stable biochemical and coagulation profiles under −80 °C and 4 °C (Figures 2, 3) for 45 days. No significant changes were observed in the biochemical parameters, and PT and aPTT remained unchanged. However, a modest but statistically significant decrease in fibrinogen concentration was observed on Day 30 (*p* = 0.0005) and Day 45 (*p* = 0.02) during storage at 4 °C. The activities of all evaluated coagulation factors showed no significant reductions. A mild decrease in antithrombin activity was observed on Day 10 and Day 30 under both −80 °C (*p* = 0.03, *p* < 0.0001, respectively) and 4 °C (*p* = 0.01, *p* = 0.0001, respectively) conditions. However, the protein C activity and thromboelastography parameters remained unchanged (Tables 3–5).

**Table 3:** Analysis of changes in plasma composition over 45 days of storage at −80 °C.

Variables	Post-lyophilization	Day 10	Day 30	Day 45
Total protein (g/dL)	4.30 [3.58–4.33]	4.00 [3.65–4.25]	4.05 [3.58–4.30]	3.95 [3.63–4.43]
Albumin (g/dL)	2.35 [2.05–2.50]	2.35 [2.05–2.50]	2.25 [2.03–2.43]	2.30 [1.88–2.43]
Glucose (mg/dL)	527.0 [479.3–565.8]	519.5 [495.5–561.3]	527.5 [496.5–570.3]	531.5 [501.3–582.0]
Osmolality (mOsm/kg)	312.0 [301.3–321.0]	301.0 [295.5–313.5]	315.5 [304.0–319.0]	298.0 [295.5–309.8]
PT (sec)	8.20 [7.90–8.40]	8.10 [7.80–8.25]	8.00 [7.85–8.25]	8.20 [7.95–8.30]
aPTT (sec)	15.60 [14.35–17.70]	14.50 [14.15–15.90]	15.00 [14.70–15.90]	14.70 [13.95–16.15]
Factor II activity (%)	103.0 [86.5–118.3]	102.5 [93.5–117.0]	90.5 [84.5–109.3]	98.0 [85.3–107.0]
Factor V activity (%)	971.0 [731.0–1,323.0]	1057.0 [843.5–1,232.0]	893.5 [733.5–1,146.0]	1028.0 [907.0–1,133.0]
Factor VIII activity (%)	402.5 [218.5–478.3]	256.0 [196.0–395.5]	321.5 [237.8–413.3]	349.5 [247.0–435.0]
Factor IX activity (%)	101.0 [91.0–140.0]	131.0 [81.0–181.0]	142.0 [116.0–174.0]	95.0 [76.0–108.0]
Factor X activity (%)	127.5 [120.0–154.3]	140.5 [125.0–185.3]	142.5 [112.0–191.0]	155.5 [128.8–172.5]
Factor XII activity (%)	64.0 [38.5–103.0]	65.0 [54.0–112.0]	100.0 [83.0–113.5]	81.0 [62.0–112.0]
Fibrinogen concentration (mg/dL)	149.0 [138.5–190.5]	141.0 [131.0–186.5]	144.0 [128.0–173.0]	146.0 [131.0–173.0]
Antithrombin activity (%)	122.0 [113.0–133.5]	108.0* [98.0–121.5]	105.0**** [94.0–116.5]	108.0 [101.5–120.5]
Protein C activity (%)	23.0 [19.5–26.5]	26.0 [21.5–26.0]	26.0 [23.0–27.5]	26.0 [21.5–29.0]

Data are presented as median (IQR). *p < 0.05, ****p < 0.0001 versus post-lyophilization group.

**Table 4:** Analysis of changes in plasma composition over 45 days of storage at 4 °C.

Variables	Post-lyophilization	Day 10	Day 30	Day 45
Total protein (g/dL)	4.30 [3.58–4.33]	4.20 [3.68–4.30]	4.10 [3.58–4.20]	4.10 [3.68–4.33]
Albumin (g/dL)	2.35 [2.05–2.50]	2.30 [2.03–2.53]	2.30 [1.98–2.50]	2.35 [1.98–2.50]
Glucose (mg/dL)	527.0 [479.3–565.8]	531.5 [476.0–577.0]	528.0 [488.0–587.5]	548.0 [511.3–608.8]
Osmolality (mOsm/kg)	312.0 [301.3–321.0]	300.5 [290.0–316.5]	310.0 [304.0–318.0]	297.5 [293.5–307.8]
PT (sec)	8.20 [7.90–8.40]	8.20 [7.90–8.40]	8.00 [7.90–8.15]	8.30 [8.05–8.45]
aPTT (sec)	15.60 [14.35–17.70]	15.30 [14.35–16.10]	15.10 [14.80–15.95]	14.50 [13.60–14.95]
Factor II activity (%)	103.0 [86.5–118.3]	103.5 [94.3–114.3]	98.0 [80.3–115.3]	99.5 [88.3–105.3]
Factor V activity (%)	971.0 [731.0–1,323.0]	917.5 [797.3–1,261.0]	893.5 [762.3–1,112.0]	962.0 [735.5–1,224.0]
Factor VIII activity (%)	402.5 [218.5–478.3]	333.0 [182.0–385.5]	356.0 [183.5–460.5]	318.0 [224.0–493.5]
Factor IX activity (%)	101.0 [91.0–140.0]	98.0 [81.0–174.0]	145.0 [70.0–169.0]	98.0 [90.0–173.0]
Factor X activity (%)	127.5 [120.0–154.3]	131.5 [119.5–156.8]	144.0 [112.8–179.5]	153.0 [125.3–185.0]
Factor XII activity (%)	64.0 [38.5–103.0]	100.0 [55.0–113.5]	94.0 [79.5–108.0]	86.0 [72.5–111.5]
Fibrinogen concentration (mg/dL)	149.0 [138.5–190.5]	140.0 [132.5–180.0]	142.0*** [124.5–170.5]	137.0* [128.5–175.0]
Antithrombin activity (%)	122.0 [113.0–133.5]	107.0* [98.5–121.0]	101.0*** [94.0–117.5]	110.0 [101.5–118.5]
Protein C activity (%)	23.0 [19.5–26.5]	25.0 [21.8–27.0]	25.5 [21.8–27.5]	24.5 [22.8–28.0]

Data are presented as median (IQR). *p < 0.05, ***p < 0.001 versus post-lyophilization group.

**Table 5:** Analysis of changes in thromboelastography parameters under different storage temperature conditions.

Variables (reference interval) (25)	Storage temperature	Post-lyophilization	Day 10	Day 30	Day 45
R time (min) [3.8 (3.6–4.3)]	−80°C	2.15 (1.33–2.38)	1.75 (1.58–2.23)	1.80 (1.58–1.90)	1.80 (1.48–2.13)
	4 °C		1.65 (1.50–1.95)	1.85 (1.69–2.03)	1.80 (1.70–2.05)
	RT		1.80 (1.63–2.00)	2.05 (1.68–2.28)	2.10 (1.88–2.33)
	38 °C		1.90 (1.80–2.20)	2.25 (2.18–2.65)	2.40 (2.20–2.43)
K time (min) [1.5 (1.2–1.6)]	−80°C	2.20 (1.83–2.43)	1.90 (1.70–2.28)	1.80 (1.70–1.90)	2.15 (2.03–2.98)
	4 °C		1.70 (1.43–1.80)	1.50 (1.18–1.90)	2.10 (1.60–2.53)
	RT		2.05 (1.73–2.23)	1.90 (1.65–2.05)	2.45 (1.90–3.15)
	38 °C		1.90 (1.53–2.23)	1.75 (1.58–1.90)	2.75 (2.25–3.30)
Alpha angle (degree) [70.7 (49.3–71.9)]	−80°C	68.00 (65.30–70.88)	70.65 (66.88–73.93)	72.35 (70.90–74.20)	69.60 (66.43–71.98)
	4 °C		72.75 (71.75–73.83)	73.65 (70.05–77.03)	71.00 (69.68–73.65)
	RT		70.30 (67.33–72.35)	71.65 (70.78–73.80)	69.25 (66.33–72.33)
	38 °C		71.30 (67.73–74.35)	72.05 (70.83–73.66)	69.56 (67.13–72.86)
Maximum amplitude (mm) [62.3 (58.9–65.2)]	-80°C	52.50 (50.65–54.58)	54.40 (52.78–57.40)	55.15 (54.15–56.88)	52.50 (47.38–54.33)
	4 °C		56.25 (55.60–58.55)	57.15 (54.60–61.08)	51.50 (50.83–56.03)
	RT		53.35 (52.30–55.90)	53.75 (52.90–55.48)	49.40 (46.10–52.30)
	38 °C		53.20 (49.03–55.70)	49.30 (46.08–53.73)	45.35* (42.46–48.03)

Data are presented as median (IQR). *p < 0.05 versus post-lyophilization group.

RT (room temperature).

#### 3.2.2 Stability evaluation over 45 days of storage at room temperature

**Figure 4:**
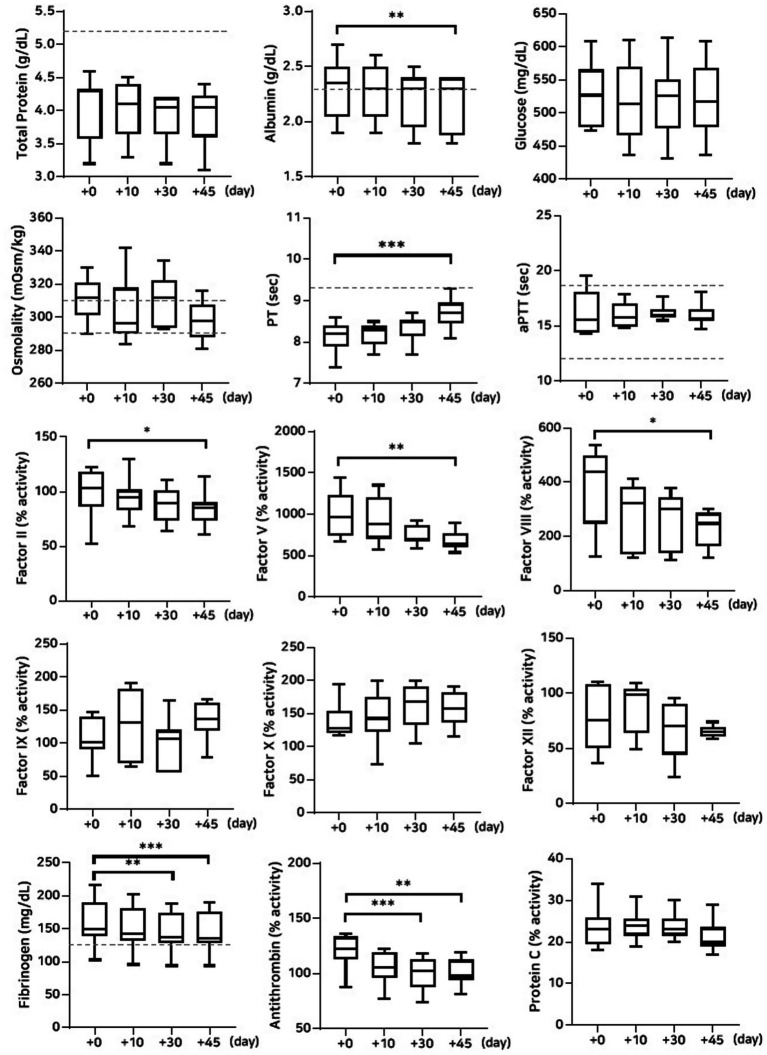
Analysis of changes in plasma composition over 45 days of storage at room temperature (*n* = 10 samples per time point). Friedman test analysis of biochemical and coagulation parameters over 45 days of storage at room temperature. The dotted lines represent the upper reference limit for PT, the upper and lower reference limits for osmolality and aPTT, and the lower reference limits for total protein, albumin, and fibrinogen concentrations. *p < 0.05, **p < 0.01, ***p < 0.001, and ****p < 0.0001.

A significant decrease in albumin concentration was observed on Day 45 (*p* = 0.008) for the group stored at room temperature. This was accompanied by a significant reduction in fibrinogen concentration on Days 30 (*p* = 0.001) and 45 (*p* = 0.0008). The activities of coagulation factors II (*p* = 0.02), V (*p* = 0.001), and VIII (*p* = 0.02) were significantly reduced on Day 45. In contrast, no significant changes were observed for factors IX, X, and XII. Antithrombin activity declined significantly on Days 30 (*p* = 0.0001) and 45 (*p* = 0.004), which is consistent with findings under other storage conditions. However, no significant changes were observed in protein C activity or thromboelastography parameters (Figure 4; Tables 5, 6).

**Table 6:** Analysis of changes in plasma composition over 45 days of storage at room temperature.

Variables	Post-lyophilization	Day 10	Day 30	Day 45
Total protein (g/dL)	4.30 [3.58–4.33]	4.10 [3.65–4.40]	4.05 [3.65–4.20]	4.05 [3.60–4.23]
Albumin (g/dL)	2.35 [2.05–2.50]	2.30 [2.05–2.50]	2.30 [1.95–2.40]	2.30** [1.88–2.40]
Glucose (mg/dL)	527.0 [479.3–565.8]	514.0 [465.8–570.3]	525.5 [476.5–550.5]	517.5 [478.3–567.5]
Osmolality (mOsm/kg)	312.0 [301.3–321.0]	296.5 [290.0–317.8]	312.0 [293.8–322.3]	297.5 [287.8–307.8]
PT (sec)	8.20 [7.90–8.40]	8.30 [7.95–8.40]	8.50 [8.15–8.55]	8.70*** [8.45–8.95]
aPTT (sec)	15.60 [14.35–17.70]	15.80 [14.90–17.00]	16.00 [15.80–16.50]	16.40 [15.50–16.50]
Factor II activity (%)	103.0 [86.5–118.3]	95.0 [83.0–101.8]	89.5 [73.0–101.0]	85.0* [73.5–90.0]
Factor V activity (%)	971.0 [731.0–1,323.0]	882.5 [705.8–1,203.0]	701.0 [676.0–867.0]	645.5** [600.0–767.3]
Factor VIII activity (%)	402.5 [218.5–478.3]	322.0 [134.5–381.5]	301.0 [138.5–343.0]	248.0* [166.0–286.5]
Factor IX activity (%)	101.0 [91.0–140.0]	132.0 [70.0–183.0]	107.0 [55.0–120.0]	137.0 [119.0–161.0]
Factor X activity (%)	127.5 [120.0–154.3]	142.5 [122.0–174.8]	168.5 [133.5–190.3]	158.0 [135.8–183.0]
Factor XII activity (%)	64.0 [38.5–103.0]	99.0 [63.5–104.0]	70.0 [44.5–90.0]	65.0 [60.5–69.5]
Fibrinogen concentration (mg/dL)	149.0 [138.5–190.5]	143.0 [132.0–181.5]	137.0** [128.5–174.0]	136.0*** [128.0–176.0]
Antithrombin activity (%)	122.0 [113.0–133.5]	106.0 [96.0–119.0]	102.0*** [87.5–113.0]	98.0** [94.5–112.5]
Protein C activity (%)	23.0 [19.5–26.5]	24.0 [21.5–25.5]	23.0 [21.5–25.5]	20.0 [19.0–23.5]

Data are presented as median (IQR). *p < 0.05, **p < 0.01, ***p < 0.001 versus post-lyophilization group.

**Table 7:** Analysis of changes in plasma composition over 45 days of storage at 38 °C.

Variables	Post-lyophilization	Day 10	Day 30	Day 45
Total protein (g/dL)	4.30 [3.58–4.33]	4.15 [3.65–4.23]	4.05 [3.53–4.30]	4.20 [3.70–4.50]
Albumin (g/dL)	2.35 [2.05–2.50]	2.30 [1.88–2.43]	2.15** [1.78–2.40]	2.15*** [1.88–2.30]
Glucose (mg/dL)	527.0 [479.3–565.8]	506.0 [465.3–539.5]	497.0**** [434.5–541.3]	488.0** [434.8–550.5]
Osmolality (mOsm/kg)	312.0 [301.3–321.0]	295.0 [289.8–317.5]	300.0 [295.0–312.3]	290.5* [285.8–297.5]
PT (sec)	8.20 [7.90–8.40]	9.00 [8.75–9.15]	10.90** [10.05–11.10]	11.70**** [10.55–11.95]
aPTT (sec)	15.60 [14.35–17.70]	18.30 [16.85–19.45]	21.80** [19.75–23.75]	21.80*** [19.75–24.75]
Factor II activity (%)	103.0 [86.5–118.3]	81.0 [74.8–85.0]	61.5** [53.8–66.0]	48.0**** [43.8–55.8]
Factor V activity (%)	971.0 [731.0–1,323.0]	678.0 [611.0–929.0]	371.0* [297.0–432.0]	312.0*** [287.0–344.0]
Factor VIII activity (%)	402.5 [218.5–478.3]	183.0 [140.3–249.5]	122.5* [99.3–163.0]	119.0* [90.0–150.0]
Factor IX activity (%)	101.0 [91.0–140.0]	81.5 [49.0–93.5]	44.5** [39.0–51.5]	49.0 [42.0–61.3]
Factor X activity (%)	127.5 [120.0–154.3]	131.0 [114.5–151.3]	77.5 [61.8–86.3]	55.5*** [51.8–67.8]
Factor XII activity (%)	64.0 [38.5–103.0]	69.0 [61.0–83.5]	30.0 [26.0–32.5]	24.0** [17.0–25.5]
Fibrinogen concentration (mg/dL)	149.0 [138.5–190.5]	137.0 [128.0–183.5]	119.0** [115.0–131.5]	112.0**** [107.0–132.5]
Antithrombin activity (%)	122.0 [113.0–133.5]	97.0 [90.0–111.0]	84.0**** [71.0–89.0]	78.0*** [74.0–90.0]
Protein C activity (%)	23.0 [19.5–26.5]	20.0 [19.0–23.0]	15.0* [14.0–17.0]	12.0**** [11.5–14.5]

Data are presented as median (IQR). *p < 0.05, **p < 0.01, ***p < 0.001, ****p < 0.0001 versus post-lyophilization group.

#### 3.2.3 Stability evaluation over 45 days of storage at 38 °C

**Figure 5:**
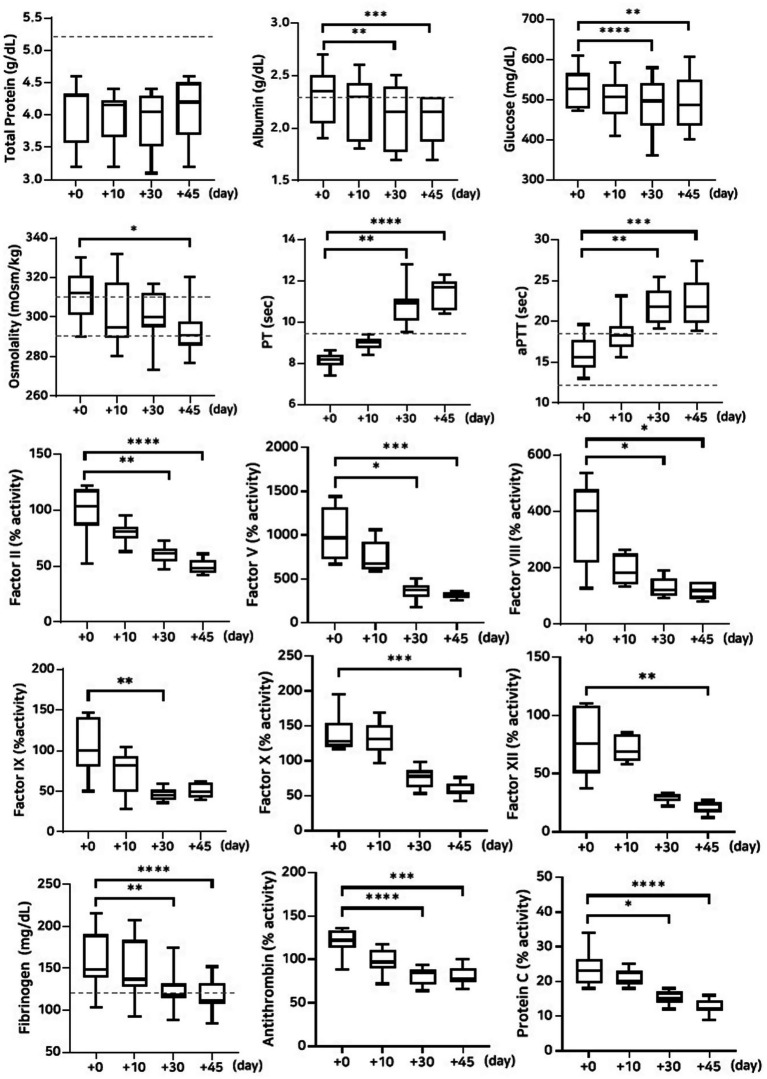
Analysis of changes in plasma composition over 45 days of storage at 38 °C (*n* = 10 samples per time point). Friedman test analysis of biochemical and coagulation parameters over 45 days of storage at 38 °C. The dotted lines represent the upper reference limit for PT, the upper and lower reference limits for osmolality and aPTT, and the lower reference limits for total protein, albumin, and fibrinogen concentrations. *p < 0.05, **p < 0.01, ***p < 0.001, and ****p < 0.0001.

The concentrations of albumin and glucose significantly decreased on Days 30 and 45 in the group stored at 38 °C (albumin: *p* = 0.002 and *p* = 0.0008; glucose: *p* < 0.0001 and *p* = 0.003, respectively). PT and aPTT were significantly prolonged (PT: *p* = 0.003 and *p* < 0.0001; aPTT: *p* = 0.001 and *p* = 0.0004), and fibrinogen concentrations significantly decreased (*p* = 0.002 and *p* < 0.0001) on Days 30 and 45, respectively. The activities of all measured coagulation factors decreased by more than 50% on Day 45 from the post-lyophilization values. The activities of antithrombin and protein C significantly decreased on Days 30 and 45, respectively (antithrombin: *p* < 0.0001 and *p* = 0.0008; protein C: *p* = 0.01 and *p* < 0.0001). Thromboelastography revealed a significant decrease in MA (*p* = 0.02) on Day 45, likely attributable to the marked loss of fibrinogen concentration (Figure 5; Tables 5, 7).

No bacterial growth was observed in any reconstituted plasma samples under aerobic culture conditions throughout the study, across all storage temperatures and time points.

### 3.3 Effect of reconstitution volume on plasma composition and osmolality

**Figure 6:**
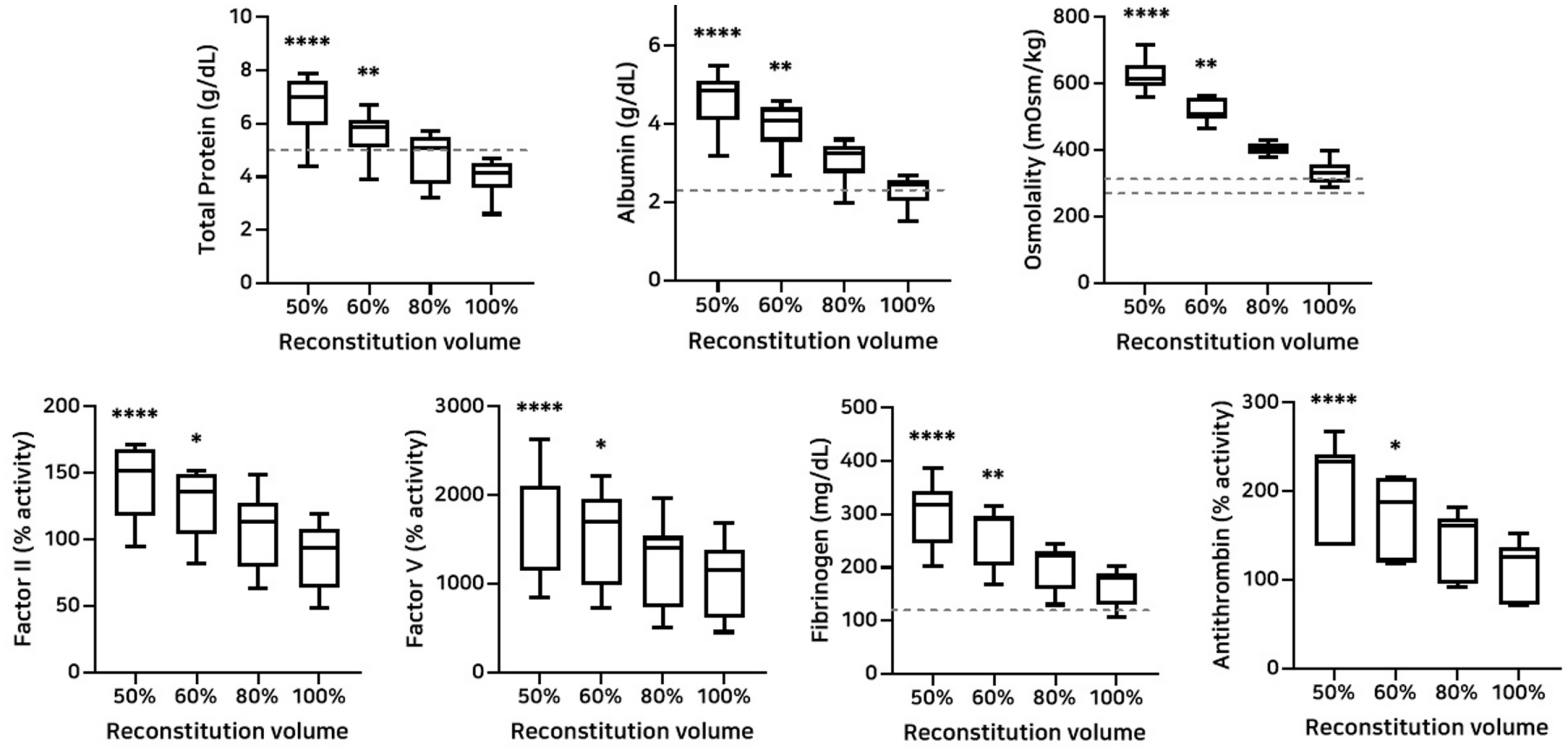
Analysis of changes in plasma composition according to reconstitution volume (*n* = 10 samples per time point). Friedman test analysis of plasma parameters according to reconstitution volume. Data are presented as median (IQR). The dotted lines represent the upper and lower reference limits for osmolality, and the lower reference limits for total protein, albumin, and fibrinogen concentrations. The 50 and 60% reconstitution groups exhibited statistically significant increases in all measured parameters compared to the 100% reconstitution group. *p < 0.05, **p < 0.01, ***p < 0.001, and ****p < 0.0001 versus 100% reconstitution group.

The effects of different reconstitution volumes on osmolality and plasma composition were determined by assessing total protein, albumin, osmolality, coagulation factors II and V activities, fibrinogen concentration, and antithrombin activity (Figure 6). Reconstitution at 50 and 60% volumes, relative to 100%, significantly increased the concentrations of total protein, albumin, osmolality, and fibrinogen concentration, as well as the activities of coagulation factors II, V, and antithrombin (Table 8).

**Table 8:** Analysis of changes in plasma composition according to reconstitution volume.

Variables	50% volume	60% volume	80% volume	100% volume
Total protein (g/dL)	7.00**** [5.95–7.63]	5.85** [5.13–6.15]	5.10 [3.75–5.50]	4.15 [3.58–4.53]
Albumin (g/dL)	4.85**** [4.10–5.13]	3.55** [4.10–4.43]	3.25 [2.75–3.45]	2.50 [2.03–2.50]
Osmolality (mOsm/kg)	616.0**** [594.8–656.5]	508.5** [496.5–557.8]	408.5 [388.8–420.0]	333.5 [303.8–359.0]
Factor II activity (%)	151.5**** [117.8–168.0]	136.5* [104.0–149.0]	113.0 [79.5–127.8]	94.0 [64.0–108.5]
Factor V activity (%)	2,083.0**** [1,147.0–2,110.0]	1,707.0* [980.5–1,961.0]	1,401.0 [733.5–1,541.0]	1,154.0 [622.5–1,388.0]
Fibrinogen concentration (mg/dL)	319.0**** [245.0–345.0]	290.0** [203.0–297.0]	222.0 [159.0–232.0]	181.0 [129.0–189.5]
Antithrombin activity (%)	234.0**** [139.0–242.0]	188.0* [120.0–215.0]	161.0 [96.0–169.0]	126.0 [72.0–137.0]

Data are presented as median (IQR). *p < 0.05, **p < 0.01, ****p < 0.0001 versus 100% reconstitution group.

## 4 Discussion

This study demonstrated that lyophilization did not significantly affect the compositional integrity of canine plasma. Storage stability analysis showed that plasma maintained stable biochemical and coagulation profiles for up to 45 days under −80 °C and 4 °C conditions. In contrast, storage at 38 °C resulted in marked deterioration of coagulation function, including prolonged PT and aPTT, significant reduction in fibrinogen concentrations, and more than 50% reductions in all measured coagulation factor activities. Room temperature storage significantly reduced the activities of coagulation factors II, V, and VIII by Day 45. However, PT, aPTT, and thromboelastography parameters remained within normal limits, suggesting acceptable short-term clinical efficacy under ambient conditions. Reconstitution at reduced volumes resulted in dose-dependent increments in albumin concentration, osmolality, and coagulation protein concentrations. These findings support the feasibility of tailoring plasma osmolarity to specific clinical needs and highlight the potential for developing customized formulations based on therapeutic goals.

Previous studies have reported promising results on the hemostatic efficacy of LP under specific conditions. For example, reconstituted canine LP maintained hemostatic capacity for up to 14 days in refrigerated storage (8). However, comprehensive evaluations of its stability across a wider range of storage temperatures and durations have not been performed. Several studies in human medicine have focused on the long-term stability of LP under various temperature settings (4, 5, 7). These studies have identified fibrinogen and factor V as the most temperature-sensitive components. Significant degradation of these factors has been reported after 6–12 months of storage at 4 °C, 25 °C, and 40 °C (4). Up to 46% fibrinogen loss has been observed after 24 months at room temperature (5). Fibrinogen concentrations remained within normal limits during refrigerated storage, but complete degradation occurred within 12 days at 56 °C. Progressive degradation of coagulation proteins, especially after storage at ≥ 30 °C for more than 4 weeks, was observed during a short-term study (1–6 weeks) using storage temperatures of 22 °C, 30 °C, and 38 °C (7).

The present study demonstrated temperature-dependent degradation of specific coagulation proteins and substantiated the existing evidence that elevated storage temperatures accelerate the degradation of plasma components. These results highlight the critical importance of selecting appropriate storage conditions to preserve the functional integrity of lyophilized plasma and support its potential clinical application in veterinary emergency care under well-controlled temperature settings.

Selective administration of LP based on patient-specific indications is warranted. For example, caution is required for patients with hemophilia A due to the temperature-sensitive degradation of coagulation factor VIII. In the present study, factor VIII activity remained stable only when stored at −80 °C or 4 °C for the 45-day observation period. Therefore, LP stored at temperatures exceeding standard refrigeration cannot be considered suitable for the treatment of hemophilia A. This highlights the importance of evaluating the stability of individual coagulation factors when determining the clinical suitability of LP.

Additionally, substantial inter-individual variability was observed across several parameters. This likely reflects biological variation in plasma composition among individual donor dogs. Greater variability may be expected when using heterogenous canine donor populations with diverse breeds, ages, and health statues, which could further affect the consistency and standardization of LP products in clinical applications.

The clinical data on LP are limited in small animal medicine. However, its potential *in vivo* efficacy has been demonstrated in several animal model studies. In swine models, LP exhibited resuscitative effects comparable to those of FFP, with similar outcomes in serum lactate concentrations, interleukin-6 levels, hemodynamic parameters, and overall hemostatic function (11, 12). In rodent models of pneumonia and lung injury, LP improved vascular permeability and attenuated inflammatory responses, mirroring the effects of FFP (13). Furthermore, in a canine hemorrhagic shock model, LP preserved the endothelial glycocalyx more effectively than isotonic saline and synthetic colloids, with protective effects comparable to FFP (14). These findings support the potential utility of LP in clinical practice for dogs.

LP has potential clinical advantages beyond its storage and handling benefits. It can be used as a hyperosmotic resuscitation agent, allows rapid administration in emergency settings, and enables timely hemostatic resuscitation. It can be used in euvolemic patients without the risk of volume overload. Volume-dependent increases in plasma component concentrations with decreasing reconstitution volumes have been reported for LP in previous *in vitro* studies in canine and human models (15, 16), and similar findings were observed in the present study. Although these effects have been well documented in vitro, *in vivo* evaluation in dogs remains limited. Notably, in vivo studies using a swine hemorrhage model demonstrated that both 100 and 50% reconstituted LP resulted in comparable improvements in hemodynamic parameters, thromboelastography profiles, and serum lactate concentrations (11, 12, 17–22). These findings suggest that hyperosmotic LP may support physiological recovery even at reduced volumes and could serve as an effective resuscitative fluid for low-volume hypotensive hemostatic resuscitation in veterinary clinical practice. Considering the diversity of clinical conditions, adjusting the osmolality of lyophilized plasma according to the veterinary patient’s volume status is important. In euvolemic patients with coagulopathy, hyperosmotic LP may allow adequate hemostatic resuscitation while minimizing fluid overload. In contrast, for hypovolemic patients, reconstituting LP with a larger volume of diluent may provide the benefits of both effective volume expansion and adequate coagulation support.

This study has several limitations. First, the relatively small sample size may limit the statistical power and generalizability of the findings. Second, the 45 days of storage may not adequately reflect long-term stability or delayed degradation of plasma components. Third, the evaluation of LP stability and efficacy was limited by the absence of additional coagulation parameters, such as von Willebrand factor and factors VII and XIII, as well as colloid osmotic pressure. Given that hyperoncotic solutions exert greater physiological effects than iso-oncotic solutions (23, 24), this should be considered in future studies. In addition, not all parameters such as fibrinogen were measured across all experimental conditions, which may affect data completeness and comparability. Fourth, several aspects of the preparation process—most notably the use of a vial-based lyophilization system—reflect experimental rather than clinically applicable conditions, and the reliance on human-derived calibrators in the absence of canine-specific alternatives may have introduced interspecies variability and affected measurement accuracy. Fifth, the interpretation of certain functional assay results should be approached with caution. For example, some thromboelastography parameters showed numerically enhanced coagulation profiles after storage, despite no statistical significance, which may reflect the confounding influence of endogenous coagulation factors present in the healthy donor whole blood used for reconstitution and potentially mask LP-specific deficiencies. Finally, because this study was conducted entirely *in vitro*, further investigation is required to support clinical translation. Future studies should include *in vivo* evaluations in dogs to assess the clinical efficacy and safety of LP under practical conditions.

In conclusion, this study demonstrated that the lyophilization process does not significantly alter the major components of canine plasma. Short-term stability was maintained even with storage at room temperature. However, storage temperature influenced protein stability. These findings emphasize the importance of selecting appropriate storage conditions to preserve product integrity. Based on the properties confirmed in this study, LP may be practical and efficient for rapid intervention in emergencies in clinical practice.

## Data Availability

The original contributions presented in the study are included in the article/supplementary material, further inquiries can be directed to the corresponding author.

## References

[1] Feuerstein SJ Skovmand K Møller AM Wildgaard K. Freeze-dried plasma in major haemorrhage: a systematic review. Vox Sang. (2020) 115:263–74. doi: 10.1111/vox.12898, PMID: 32090336

[2] Glassberg E Nadler R Gendler S Abramovich A Spinella PC Gerhardt RT. Freeze-dried plasma at the point of injury: from concept to doctrine. Shock. (2013) 40:444–50. doi: 10.1097/shk.0000000000000047, PMID: 24089000

[3] Bakaltcheva I O'Sullivan AM Hmel P Ogbu H. Freeze-dried whole plasma: evaluating sucrose, trehalose, sorbitol, mannitol and glycine as stabilizers. Thromb Res. (2007) 120:105–16. doi: 10.1016/j.thromres.2006.07.005 16962645

[4] Zur M Glassberg E Gorenbein P Epstein E Eisenkraft A Misgav M. Freeze-dried plasma stability under prehospital field conditions. Transfusion. (2019) 59:3485–90. doi: 10.1111/trf.15533, PMID: 31568580

[5] Bux J Dickhorner D Scheel E. Quality of freeze-dried (lyophilized) quarantined single-donor plasma. Transfusion. (2013) 53:3203–9. doi: 10.1111/trf.12191 23581390

[6] Peng HT Moes K Singh K Rhind SG Pambrun C Jenkins C. Post-reconstitution hemostatic stability profiles of Canadian and German freeze-dried plasma. Life. (2024) 14:172. doi: 10.3390/life14020172, PMID: 38398681 PMC10890410

[7] Jennings I Kitchen DP Woods TAL Kitchen S Preston FE Walker ID. Stability of coagulation proteins in lyophilized plasma. Int J Lab Hematol. (2015) 37:495–502. doi: 10.1111/ijlh.12318, PMID: 25496193

[8] Edwards TH Meledeo MA Peltier GC Ruiz DD Henderson AF Travieso S. Effects of refrigerated storage on hemostatic stability of four canine plasma products. Am J Vet Res. (2020) 81:964–72. doi: 10.2460/ajvr.81.12.964, PMID: 33251844

[9] Martinaud C Civadier C Ausset S Verret C Deshayes A-V Sailliol A. In vitro hemostatic properties of French lyophilized plasma. Anesthesiology. (2012) 117:339–46. doi: 10.1097/ALN.0b013e3182608cdd, PMID: 22739764

[10] Goggs R Brainard B de Laforcade AM Flatland B Hanel R McMichael M. Partnership on rotational viscoelastic test standardization (Provets): evidence-based guidelines on rotational viscoelastic assays in veterinary medicine. J Vet Emerg Crit Care. (2014) 24:1–22. doi: 10.1111/vec.12144, PMID: 24422679

[11] McCully SP Martin DT Cook MR Gordon NT McCully BH Lee TH. Effect of ascorbic acid concentrations on hemodynamics and inflammation following lyophilized plasma transfusion. J Trauma Acute Care Surg. (2015) 79:30–8. doi: 10.1097/ta.0000000000000684, PMID: 26091311

[12] Shuja F Shults C Duggan M Tabbara M Butt MU Fischer TH. Development and testing of freeze-dried plasma for the treatment of trauma-associated coagulopathy. J Trauma. (2008) 65:975–85. doi: 10.1097/TA.0b013e3181801cd9, PMID: 19001961

[13] Pati S Peng Z Wataha K Miyazawa B Potter DR Kozar RA. Lyophilized plasma attenuates vascular permeability, inflammation and lung injury in hemorrhagic shock. PLoS One. (2018) 13:e0192363. doi: 10.1371/journal.pone.0192363 29394283 PMC5796727

[14] Ryan MA Ford R Ewer N Hall KE Guillaumin J Edwards TH. Sidestream dark field video microscopy demonstrates shelf-stable blood products preserve the endothelial glycocalyx in a canine hemorrhagic shock model. Am J Vet Res. (2024) 85:ajvr.24.05.0152. doi: 10.2460/ajvr.24.05.0152 39389101

[15] Edwards TH Meledeo MA Peltier GC Henderson AF Pompa LA Bynum JA. In vitro evaluation of hyperosmotic canine plasma suitable for infusion. J Vet Emerg Crit Care. (2024) 34:63–8. doi: 10.1111/vec.13353, PMID: 37966879

[16] Iapichino GE Ponschab M Cadamuro J Süssner S Gabriel C Dieplinger B. Concentrated lyophilized plasma used for reconstitution of whole blood leads to higher coagulation factor activity but unchanged thrombin potential compared with fresh-frozen plasma. Transfusion. (2017) 57:1763–71. doi: 10.1111/trf.14123, PMID: 28439902

[17] Lee TH McCully SP McCully BH Sands C Hampton DA Louis SG. Comparison of the hemostatic efficacy of low-volume lyophilized plasma reconstituted using sterile water, lactated ringer’s, normal saline, and Hextend solutions. J Trauma Acute Care Surg. (2014) 76:264–1; (discussion 71–2). doi: 10.1097/ta.0000000000000109 24458032

[18] McCully SP Lee TH McCully BH Sands CL Rick EA Dean RK. Reconstitution fluid type does not affect pulmonary inflammation or DNA damage following infusion of lyophilized plasma. J Trauma Acute Care Surg. (2015) 78:231–7; (discussion 7–9). doi: 10.1097/ta.0000000000000524 25757106

[19] Spoerke N Zink K Cho SD Differding J Muller P Karahan A. Lyophilized plasma for resuscitation in a swine model of severe injury. Arch Surg. (2009) 144:829–34. doi: 10.1001/archsurg.2009.154 19797107

[20] Lee TH Watson K Fabricant L Barton J Differding J Kremenevskiy I. Hyperosmolar reconstituted lyophilized plasma is an effective low-volume hemostatic resuscitation fluid for trauma. J Trauma Acute Care Surg. (2013) 75:369–75. doi: 10.1097/TA.0b013e31829bb67c, PMID: 23928743

[21] Lee TH Van PY Spoerke NJ Hamilton GJ Cho SD Watson K. The use of lyophilized plasma in a severe multi-injury pig model. Transfusion. (2013) 53:72S–9S. doi: 10.1111/trf.12039 23301977

[22] Dufour-Gaume F Cardona V Bordone A Montespan F Vest P Legland A-M. Efficacy and safety of novel freeze-dried plasma products in a porcine combat casualty model. Transfusion. (2024) 64:1670–82. doi: 10.1111/trf.17971, PMID: 39121435

[23] Mendes RS Oliveira MV Padilha GA Rocha NN Santos CL Maia LA. Effects of crystalloid, hyper-oncotic albumin, and iso-oncotic albumin on lung and kidney damage in experimental acute lung injury. Respir Res. (2019) 20:155. doi: 10.1186/s12931-019-1115-x, PMID: 31311539 PMC6636113

[24] Boldt J Ducke M Kumle B Papsdorf M Zurmeyer EL. Influence of different volume replacement strategies on inflammation and endothelial activation in the elderly undergoing major abdominal surgery. Intensive Care Med. (2004) 30:416–22. doi: 10.1007/s00134-003-2110-7 14712346

[25] Cho S Kang M Jung R Kim M Cha S Shin C. Thromboelastographic evaluation in dogs with hyperadrenocorticism. J Biomed Transl Res. (2023) 24:151–61. doi: 10.12729/jbtr.2023.24.4.151

